# CARM1 automethylation is controlled at the level of alternative splicing

**DOI:** 10.1093/nar/gkt415

**Published:** 2013-05-30

**Authors:** Lu Wang, Purin Charoensuksai, Nikole J. Watson, Xing Wang, Zibo Zhao, Carlos G. Coriano, Leslie R. Kerr, Wei Xu

**Affiliations:** ^1^McArdle Laboratory for Cancer Research, University of Wisconsin School of Medicine and Public Health, 1400 University Avenue, Madison, WI 53706, USA and ^2^Department of Biology, Life & Health Sciences Building D242, Trent University, Peterborough ON K9J 7B8, Canada

## Abstract

Co-activator-associated arginine methyltransferase 1 (CARM1) is subjected to multiple post-translational modifications. Our previous finding that automethylation of CARM1 is essential for regulation of transcription and pre-mRNA splicing prompted us to investigate how automethylation is regulated. Here, we report that automethylation is regulated by alternative splicing of CARM1 mRNA to remove exon 15, containing the automethylation site. Specifically, we find that two major alternative transcripts encoding full-length CARM1 (CARM1FL) and CARM1 with exon 15 deleted (CARM1ΔE15) exist in cells, and each transcript produces the expected protein. Further biochemical characterizations of the automethylation-defective mutant and CARM1ΔE15 reveal overlapping yet different properties. Interestingly, other arginine methylation substrates also have missing exons encompassing the site(s) of methylation, suggesting that protein arginine methylation level may, in general, be controlled by the alternative splicing mechanism. Finally, we observed differential distribution of CARM1FL and CARM1ΔE15 in epithelial and stromal cells in normal mouse mammary gland. Thus, alternative splicing not only serves as the determinant for CARM1 automethylation but also generates cell type-specific isoforms that might regulate normal ERα biology in the mammary gland.

## INTRODUCTION

Co-activator-associated arginine (R) methyltransferase 1 (CARM1), also known as PRMT4, belongs to the type I protein arginine methyltransferase (PRMT) family that asymmetrically dimethylates protein substrates on arginines (1). CARM1 was originally identified as a p160 family GRIP1-interacting protein in a yeast two-hybrid screen (2). CARM1 is involved in the transcriptional activation of cancer-relevant transcription factors, including NF-κB, p53, E2F1 and steroid receptors, among which activation of estrogen receptor α (ERα) is best characterized (3). CARM1 has a variety of protein substrates, making it a multifunctional protein engaged in diversified cellular processes. For instance, CARM1 methylates histone H3 at R2, R17 and R26 (4), which correlates with activation of ER-target gene pS2 (5). In addition, CARM1 methylates a number of non-histone proteins, including transcription co-factor CBP/p300, RNA-binding proteins HuR and HuD, splicing factors, as well as poly-A-binding protein 1 (PABP1) (6). Importantly, loss of CARM1 in the mouse embryo leads to abrogation of the estrogen response and reduced expression of some ERα-target genes, further highlighting the functional importance of CARM1 in ERα-regulated gene expression (7). Furthermore, using a gain-of-function approach in ERα-positive breast cancer cells, we showed that 2-fold CARM1 overexpression in MCF7 cells led to growth inhibition, activation of differentiation markers and inhibition of anchorage-independent growth (8). Microarray results showed that ∼60% of 17β-estradiol (E2)-regulated genes was affected by CARM1 overexpression, suggesting that CARM1 serves as a main determinant of ERα-target gene expression (8). ERα regulates a number of genes that are essential for the etiology and progression of breast cancer. These findings suggest that CARM1 uniquely regulates growth inhibition and differentiation in ERα-positive breast cancer cells through global regulation of ERα-regulated genes.

Although the regulation of ERα-dependent transcription and biological effects by CARM1 has been studied extensively in breast cancer cells (8–10), the co-localization of CARM1 with ERα in primary breast tumors and normal mammary gland has not been well characterized. By analyzing ∼300 ERα-positive human breast tumor biopsy samples, we found that the expression level of CARM1 positively correlated with ERα level in low-grade tumors (8). The strong correlation of the expression pattern of CARM1 and ERα in breast cancer cells implicates roles of CARM1 in ERα biology. Mammary gland is a hormone-sensing organ whose morphogenesis and development depend on ERα (11). ERα is expressed in both the epithelium and stroma of mouse mammary gland (12), and epithelial ERα signaling is required for ductal elongation, side branching and alveologenesis (13). Therefore, characterization of the expression pattern of CARM1 in conjunction with ERα in normal mammary gland would provide insights into its putative function in normal mammary gland development.

During the past decade, several post-translational modifications have been identified on CARM1, each of which regulates distinct aspects of CARM1 function (14–17). CARM1 can be phosphorylated on at least three sites, two of which have been shown to regulate CARM1 enzymatic activity; phosphorylation on serine (S) 229 prevents CARM1 homodimerization (14), and phosphorylation on S217 blocks S-Adenosylmethionine (SAM) binding (15). As methyltransferase activity of CARM1 is essential for its co-activator function, a CARM1 phosphorylation mimetic mutant exhibited a marked decrease in the ability to stimulate ERα-mediated transcription (14,15). Recently, a third phosphorylation site was identified at S448, which mediates the direct interaction of CARM1 with unliganded ERα to mediate ligand-independent activation of ERα (16). Finally, using top-down mass spectrometry, we mapped a single CARM1 automethylation site to R551 (in exon 15) in recombinant mouse protein, which is conserved among all vertebrate CARM1 proteins (17). Mutation of the automethylation site from arginine to lysine does not alter the enzymatic activity of CARM1. However, both CARM1-activated ERα transcription and pre-mRNA splicing were impaired (17). This result suggests that automethylation of CARM1 plays an essential function in regulation of transcription and splicing events. Although the mass spectrometry showed that nearly 100% of the recombinant CARM1 is automethylated, whether endogenous CARM1 is automethylated and how automethylation is regulated remain unclear.

Given that automethylation of CARM1 has functional significance, we investigated the molecular mechanism and cellular signaling that could regulate CARM1 automethylation. We find that Ensembl annotates two human CARM1 transcripts, one with 16 exons and the other with 15 exons. Interestingly, the short transcript lacks exon 15, where the CARM1 automethylation site resides. Using specific primers and antibodies developed in our laboratory, both CARM1 isoforms mRNAs and the corresponding CARM1 full-length (CARM1FL) and exon 15 deleted proteins (CARM1ΔE15) can be detected in various cell lines and tissues. Furthermore, we demonstrated that CARM1FL protein expression is both necessary and sufficient for its automethylation, suggesting that endogenous CARM1 automethylation is largely determined by the production of CARM1FL protein, and ultimately depends on alternative splicing to produce the CARM1FL transcript. Further biochemical characterization of the automethylation-defective mutant and CARM1ΔE15 reveals overlapping yet different properties. Finally, we analyzed the two endogenous CARM1 isoforms, CARM1FL and CARM1ΔE15, expression patterns in normal mouse mammary gland, and we showed that both isoforms are expressed in luminal and stromal compartments. Our findings reveal an unexpected mechanism by which alternative splicing not only serves as the determinant for CARM1 automethylation but also generates cell type-specific isoforms that might regulate ERα functions in mammary gland.

## MATERIALS AND METHODS

### Cells and tissue culture

All cell lines were purchased from American Type Culture Collection (ATCC) and used within 6 months. Human breast cancer cell line MCF7, T47D, ZR75, MDA-MB-468, CAL51, SK-BR3, Hs578T, BT549 and MB231 were maintained in Dulbecco’s modified Eagle’s medium (Life Technologies, Grand Island, NY, USA) supplemented with 10% fetal bovine serum (Life Technologies), 100 U/ml penicillin and 100 µg/ml streptomycin and incubated at 37°C in a humidified atmosphere containing 5% CO_2_. Human breast cancer cell line BT474, human glioblastoma-astrocytoma, epithelial-like cell line U87 (gift from Weibo Cai) and human neuroblastoma cell line IMR32 (gift from Jeffrey Johnson) were cultured in RPMI 1640 medium. The 20 human tissue RNA samples were purchased from Life Technologies (CAT# AM6000). Each pool consists of RNA from at least three normal tissue donors.

### Animals and tissue collection

Female BALB/c mice (Charles River Laboratories, Quebec) were housed, two to four mice per cage. Mammary glands were collected at approximately postnatal day (PND) 60 (±2 PND), and only when mice were in estrus. The fourth and ninth inguinal mammary glands were fixed overnight and then underwent immunohistochemical procedures. Ovary and brain tissues were dissected from ACI strain female rat for RNA extraction.

### Expression and purification of human recombinant CARM1

The human CARM1FL and CARM1ΔE15 cDNAs were cloned to pFN21K HaloTag CMV Flexi Vector (Promega). These constructs were expressed in transiently transfected HEK293T cells, affinity-purified using HaloLink resin (Promega) and eluted by Tobacco Etch Virus (TEV) protease. The C-terminal Halo tag remains covalently coupled to the resin, leaving the purified CARM1 proteins without a tag. The exact details of expression and purification of the HaloTag CARM1 proteins were as described previously (18).

### Detection of CARM1 isoforms using reverse transcribed–polymerase chain reaction and real-time polymerase chain reaction

RNA was isolated using RNeasy kit (Qiagen) following the manufacturers’ instructions. DNA contamination was digested using DNAfree (Ambion). RNA was quantified and 2 µg was added to Superscript II (Invitrogen) reverse transcription reactions with random primer. Quantitative polymerase chain reaction (qPCR) was performed using SYBR Green MasterMix (Invitrogen). Four microliters of 1:100 diluted cDNA was added to each 20-µl reaction, and four replicates were performed per condition. Ct levels were compared with a relative standard curve of similarly prepared cDNA from the same cell type. The primer sequences used in this study were as follows: for all CARM1 variants, forward primer: 5′-CAAGGCAGGGGACACG-3′, reverse primer: 5′-TGGCTGTTGACTGCATAGTG-3′; for CARM1FL, forward primer: 5′-ATGAGCACGGGGATTGTCCAA, reverse primer: 5′-TGGCTGTTGACTGCATAGTG-3′. Primers for human pS2, IGFBP4 and PTGES were described previously (17).

### Immunoprecipitation and western blot quantification

One microgram (1 μg/μl) of whole cell lysates was generated from U87 or IMR32 cells, from which 1% (10 µl) was used as input sample. For immunoprecipitation experiment, 5 µg of methyl-E15 antibody was mixed with the cell lysates and incubated overnight at 4°C. On the second day, 35 µl of protein A beads slurry was added and incubated for 4 h. Approximately 1 ml of flow-through cell lysates was collected, from which 1% (10 µl) was used as ‘FT’ sample. The beads were washed with ice-cold phosphate-buffered saline/0.5% Triton and boiled with 100 µl of sodium dodecyl sulfate (SDS)-sample buffer, from which 10% (10 µl) was loaded on SDS–polyacrylamide gel electrophoresis (SDS–PAGE) and labeled as ‘IP’ sample. CARM1 levels detected with E16Ab in the same proportion of input, flow-through (FT) and IP samples were quantified using ImageJ.

### Luciferase reporter assay

HEK293T cells were co-transfected with CMX-ERα (5 ng), pGRIP1 (100 ng), pERE (100 ng) and 200 ng of pCMX-Flag vector control, pCMX-Flag-CARM1FL or pCMX-Flag-CARM1ΔE15, respectively, using TransIT-LT1 (Mirus Research). Renilla-luciferase expression vector (100 ng) was transfected for normalization of transfection efficiency. Cells were then treated with DMSO or 10 nM E2 for 48 h before lysis and detection of luciferase. Firefly luciferase from the UAS reporter was normalized with renilla luciferase.

### *In vitro* methylation assays

Enzymes and substrates were incubated in 15 µl of 5 mM MgCl_2_, 20 mM HEPES, pH 7.9, 1 mM ethylenediaminetetraacetic acid, 1 mM DTT and 10% glycerol containing 1 µl of ^3^H–*S*-adenosylmethionine (78 Ci/mmol, GE Healthcare) for 5 min to 1 h. The reaction mix was then separated by SDS–PAGE and fixed in methanol: acetic acid (50:5%) containing Coomassie brilliant blue for 2 h followed by destaining in Coomassie-free methanol: acetic acid for 1 h. Fixed gels were incubated in ‘Amplify’ (Amersham Biosciences) scintillation fluid for 30 min, stored at −80°C overnight and exposed to a film.

### Generation of peptide antibodies to detect various CARM1 isoforms and the endogenously automethylated CARM1

All peptide antibodies E16 (detects both isoforms of CARM1) and me-E15 (detects automethylated CARM1) were generated by Genemed Synthesis Inc., TX, USA. To generate methyl-CARM1–specific antibody (me-E15), the KLH-conjugated E15 peptide with R551 asymmetrically dimethylated was synthesized. E16 and me-E15 antibodies encompass peptide sequences TNTMHYGSASPMSIP and VNHTHSR(me2) MGSIMSTG, corresponding to human CARM1 amino acids 593–608 and 544–558, respectively, were used to immunize rabbits. To purify dimethyl-CARM1–specific antibody (me-E15), 10 mg dimethyl peptide (column A) and 10 mg control peptide (non-methyl) (column B) were coupled to cyanogen bromide-activated agarose beads separately. Hundred milliliters of antisera was then incubated with the peptide-agarose column A. The unbound antiserum was washed with 1× phosphate-buffered saline buffer. After several washes, the antibody was eluted with 0.1 M glycine, pH 2.5; neutralized with 1 M Tris, pH 8.0. The antibody was stabilized with 0.1% bovine serum albumin (elute A). Elute A was then incubated with column B and then same procedure was followed for elution. The flow through from column B was the dimethyl-specific Ab (me-E15). The elute from column B that is elute B was the control Ab (E15; detects total full-length CARM1 protein, primarily unmethylated form). The antisera, unbound fraction and purified antibody were then tested by enzyme-linked immunosorbent assay.

### Immunohistochemistry staining of CARM1 in normal mouse mammary gland

To optimize E15Ab, me-E15Ab and E16Ab for immunohistochemistry (IHC), 70% confluent normal mouse embryonic fibroblast (MEF) (MEF-CARM1+/+ and MEF derived from CARM1 null mice (MEF–CARM1−/−) were seeded on the poly-l-lysine coverslips. After 24 h, the cell pellets were fixed in 3.7% formaldehyde, paraffin embedded and subject to immunostaining. All the reagents for IHC were purchased from Biocare Medical (Concord, CA, USA). After antigen retrieval and a 30-min incubation with avidin and biotin, the cells were incubated with E16 (1:250), me-E15 (1:250) or E15 (1:250) primary antibodies, respectively, diluted in DaVinci Green for 2 h at room temperature and subsequently with the biotinylated goat anti-rabbit secondary IgG antibody and streptavidin horseradish peroxidase for 15 min each at room temperature. Finally, DAB (3,3′-diaminobenzidine) was used as substrate, and hematoxylin was used for counter-stain. Twenty-fold excess amount of blocking peptides for E15, methyl-E15 or E16 was pre-mixed with the corresponding antibodies to determine the antibody specificity.

Staining for CARM1 isoforms was performed on paraffin-embedded mammary gland sections (6 μm). After de-paraffinization, antigen retrieval and a 30-min incubation with 6% goat serum, tissue sections were incubated with E16 (1:125), me-E15 (1:275) or E15 (1:275) primary antibodies for 19 h at 4°C. After this, appropriate secondary IgG antibodies (me-E15: BA-1000; 1:150; Vector Laboratories Inc., and E16/E15 SC-2040: 1:200; Santa Cruz Biotechnologies) were applied for 1.5 h at room temperature. Pictures of representative areas within the mammary gland were taken using a Leica DM6000 B camera, and cells were counted using Photoshop©. For each antibody, tissue sections from 10–12 young adult (PND 60) mice were used, and ∼500–750 nuclei per representative area were counted, with 2000–5000 nuclei counted for each mammary gland. Counts were expressed as the percentage of cells positively stained for E16, me-E15 or E15 relative to the total number of cells in each mammary gland compartment (i.e. luminal or stromal). Statistical comparisons were performed on the ratio of luminal over stromal-positive CARM1 counts using non-parametric two-tailed Mann–Whitney U-tests as the transformed data violated parametric assumptions. The software package Statistica version 6.1 (StatSoft) was used for all analyses.

## RESULTS

### Cell and tissue-specific alternative splicing of human CARM1 transcript result in two major isoforms

**Figure 1.**
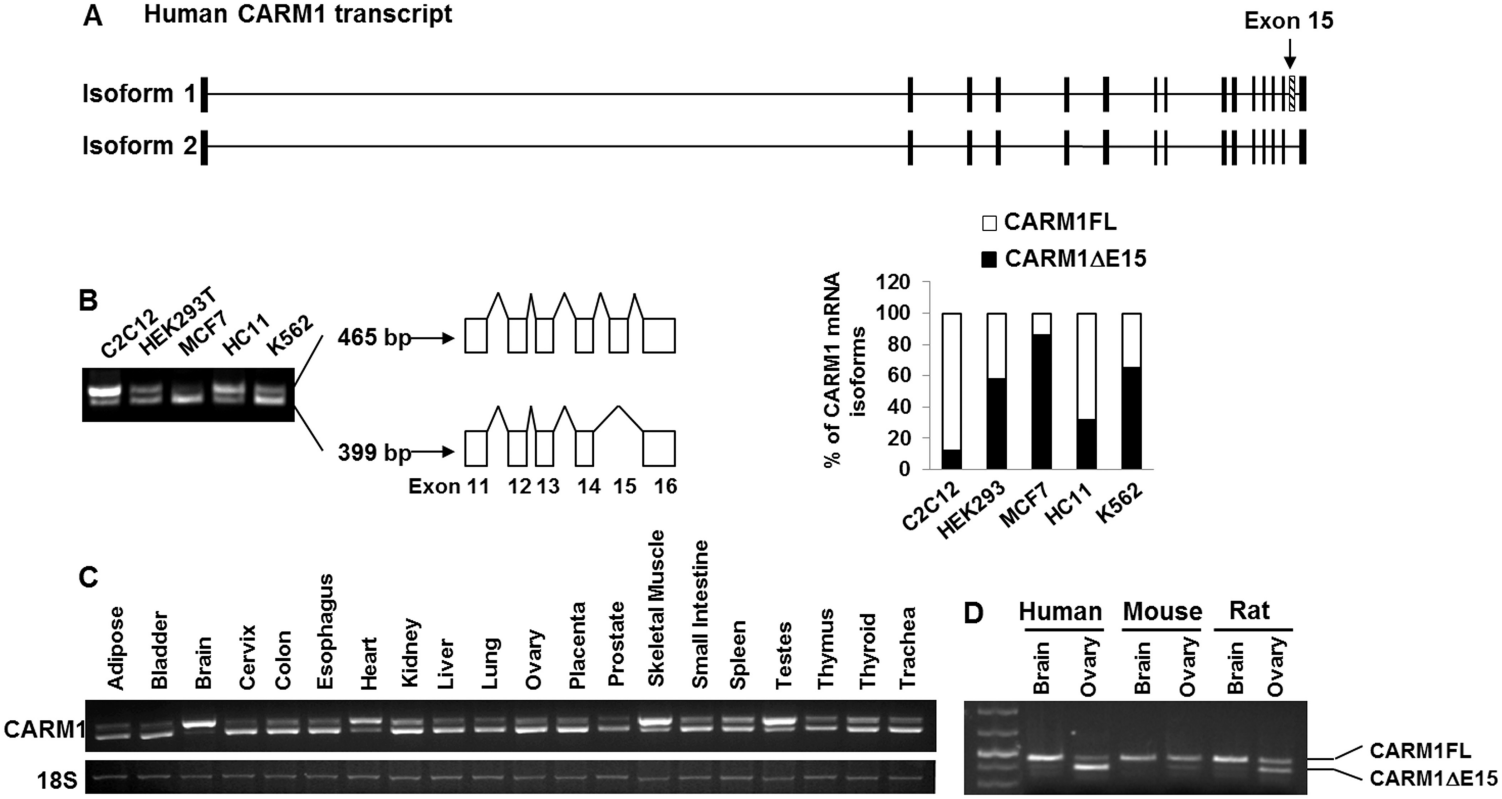
Alternative splicing of CARM1 is cell-type and tissue-specific. (**A**) Two isoforms of human CARM1 transcripts are annotated in ensembl. (**B**) Total RNAs of different cell lines were extracted, and the expression levels of CARM1 isoforms (465 and 399 bp) were detected by RT–PCR, using primers described in ‘Materials and Methods’ section (left). The relative level of CARM1 isoforms was determined by band-scan and plotted (right). (**C**) The expression pattern of CARM1 isoforms in 20 human tissue samples. RT–PCR was performed with total RNAs derived from 20 human tissue samples in triplicate, represented data were shown. The 18 S was used as a loading control. (**D**) The expression pattern of CARM1 isoforms in brain and ovary of human, mouse and ACI rat detected by RT–PCR.

Our finding that automethylation of CARM1 is essential for regulating ERα-mediated transcription (17) raises the question of how automethylation is regulated. We find that two human CARM1 transcripts are annotated by Ensembl (Figure 1A). Compared with the full-length 2968-bp transcript, the 2899-bp short transcript lacks exon 15, where the CARM1 automethylation site resides (17). A previous study reported four CARM1 isoforms in normal rat tissue cDNA library (19) differing in the C-terminus. To discern if all of these isoforms also exist in the human tissues and cell lines, we designed primers to amplify the C-terminal region between Exon 11 and Exon 16 of human CARM1 cDNA. As shown in Figure 1B, only two CARM1 isoforms could be detected in multiple human cell lines, although the expression level and relative ratio of the two isoforms varies. In keeping with our finding that the exon 15 deleted isoform is the major CARM1 transcript in MCF7 (Figure 1B), we cloned human CARM1 mRNA from MCF7 cells as cDNAs and discovered that exon 15 was indeed missing from the predicted CARM1 mRNA sequence. No deletion was found by sequencing genomic DNA, nor was any mutation identified in CARM1 mRNA derived from MCF7 cancer cells (data not shown), implying that CARM1 in cancer cells is regulated by alternative splicing, resulting in exon 15 exclusion. To examine the tissue-specific distribution of CARM1 full-length (CARM1FL) and exon 15 deleted (CARM1ΔE15) isoforms, we performed reverse transcribed (RT)–PCR of CARM1 transcripts using aforementioned primers with RNA derived from 20 normal human tissues (Figure 1C). CARM1ΔE15 was found to be the dominant form in most human tissues. CARM1FL seems to be the major isoform in only four human tissues, including brain, heart, skeletal muscle and testis. The two isoforms of CARM1 can be detected in human, mouse and rat tissues (Figure 1D), suggesting that alternative splicing is conserved among species. CARM1FL isoform is dominant in brain across species, however, interestingly, the ratio of CARM1FL/ΔE15 isoforms is the highest in mouse ovary as compared with those in rat and human (Figure 1D). This implicates that alternative splicing is likely controlled by the common *cis*-elements but varies at the level of *trans*-factors.

### Generation of antibodies for detection of CARM1 full-length protein (E15), automethylation-specific CARM1 (me-E15) and both isoforms of CARM1 (E16)

**Figure 2.**
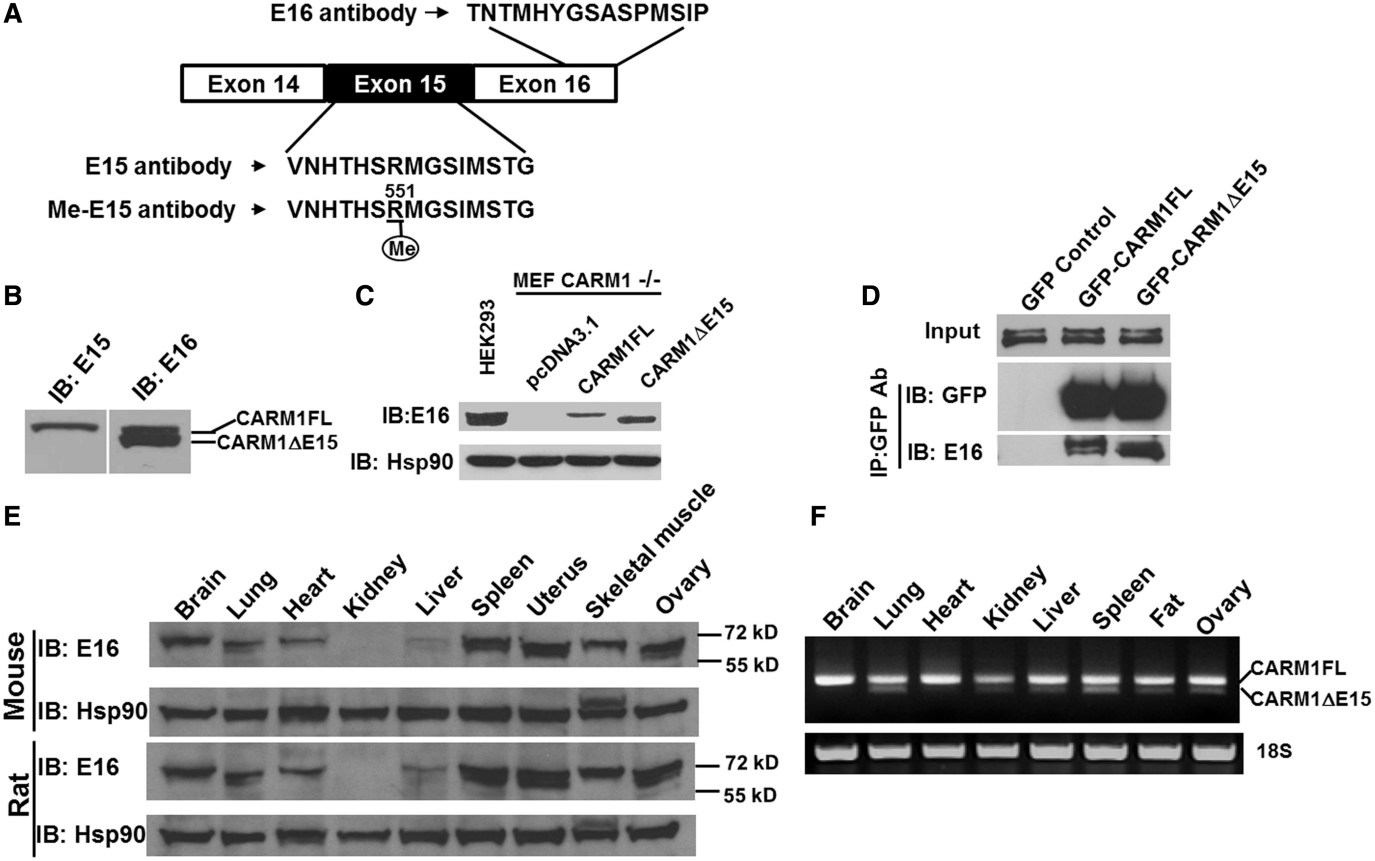
Generation of antibodies to detect full-length (E15), methylated (E15-me) and both forms of CARM1 (E16) and detection of CARM1 isoform proteins in mouse tissues. (**A**) Polyclonal peptide antibodies were generated using the annotated peptides in exon 15 or 16 or asymmetrically dimethylated exon 15 peptide. (**B**) E16 detects both the endogenous CARM1FL and the methylated CARM1ΔE15 in HEK293T cells, whereas E15 only detects CARM1FL. (**C**) E16 could detect either CARM1FL or CARM1ΔE15 in CARM1 null MEF cells transfected with either cDNA expressing plasmid. CARM1 null MEF cells were transiently transfected with cDNA expressing CARM1FL or CARM1ΔE15 for 24 h. The protein level of exogenously expressed CARM1 was detected by western blot using E16. Cell lysates from the wild-type MEF cells were used as positive control (right panel). (**D**) Plasmids expressing GFP, GFP-CARM1FL or GFP-CARM1ΔE15 were transiently transfected into HEK293T cells, respectively. After 24 h, the GFP-CARM1 fusion protein was immunoprecipitated using GFP antibody. The endogenous CARM1 in the immunoprecipitates was detected using E16 Ab on western blot. (**E**) Endogenous CARM1 isoform proteins were detected in different mouse and rat tissues by western blot using E16. (**F**) Total RNAs were extracted from the freshly prepared mouse tissues and reverse transcribed. CARM1 isoforms were amplified by PCR using primers in Figure 1B.

To examine whether proteins are generated from the transcripts, we developed peptide antibodies using the indicated sequence in Figure 2A and a methyl-specific antibody using a peptide encompassing me-R551 as the antigen. The methyl-specific antibody also allowed us to discern whether endogenous full-length CARM1 protein is automethylated. These antibodies were denoted as E16 (detects both isoforms of CARM1; CARM1FL and CARM1ΔE15), E15 (detects full length protein; CARM1FL) and me-E15 (detects automethylated CARM1), respectively. Figure 2B shows that, as expected, E16 could detect endogenous FL and ΔE15 proteins, whereas E15 only detects endogenous FL protein in HEK293 cells. The specificity of the antibodies was further confirmed with overexpression of Flag-CARM1FL and CARM1ΔE15 proteins in HEK293 cells. To confirm that the two bands ∼70 kDa recognized by E16 were indeed encoded by two CARM1 isoforms, we transfected CARM1 null MEF cells with CARM1FL and CARM1ΔE15 cDNA expression plasmids, respectively, and performed western blot using the E16 antibody. As shown in Figure 2C, E16 detects two endogenous CARM1 bands in HEK293 cells, corresponding to the position of the exogenously expressed CARM1FL and CARM1ΔE15 in CARM1 null cells. We have previously shown that CARM1 forms dimers in solution (14). To determine whether the two CARM1 splicing isoform proteins can dimerize, we transfected GFP-CARM1FL and GFP-CARM1ΔE15 expression plasmids to HEK293 cells and immunoprecipitated GFP-CARM1 fusion proteins to detect which form of the endogenous CARM1 present in HEK293 cells could be co-immunoprecipitated. As shown in Figure 2D, both CARM1FL and CARM1ΔE15 protein co-precipitated with GFP-CARM1 fusion proteins with a slight preference for homodimer, suggesting that the two CARM1 isoform proteins could form either homo- or hetero-dimers. This finding was further validated using exogenous GFP-CARM1FL and Flag-CARM1FL or Flag-CARM1ΔE15 (Supplementary Figure S1) co-transfected to HEK293 cells. These data suggest that both CARM1FL and CARM1ΔE15 could form homo- and hetero-dimers, and automethylation does not affect oligomerization. As E16 could detect both isoforms of the CARM1 protein, we examined the expression pattern of CARM1 protein isoforms across various mouse tissues. We found that endogenous CARM1 protein isoforms (Figure 2E) largely mirrors the mRNA isoforms (Figure 2F) where CARM1FL is predominant in mouse brain, heart and muscle tissues, and both CARM1 isoform proteins could be detected in mouse lung, spleen and ovary tissues (Figure 2E). Similar CARM1 isoform protein pattern was observed with rat tissues.

### Endogenous CARM1FL protein is predominately automethylated

**Figure 3.**
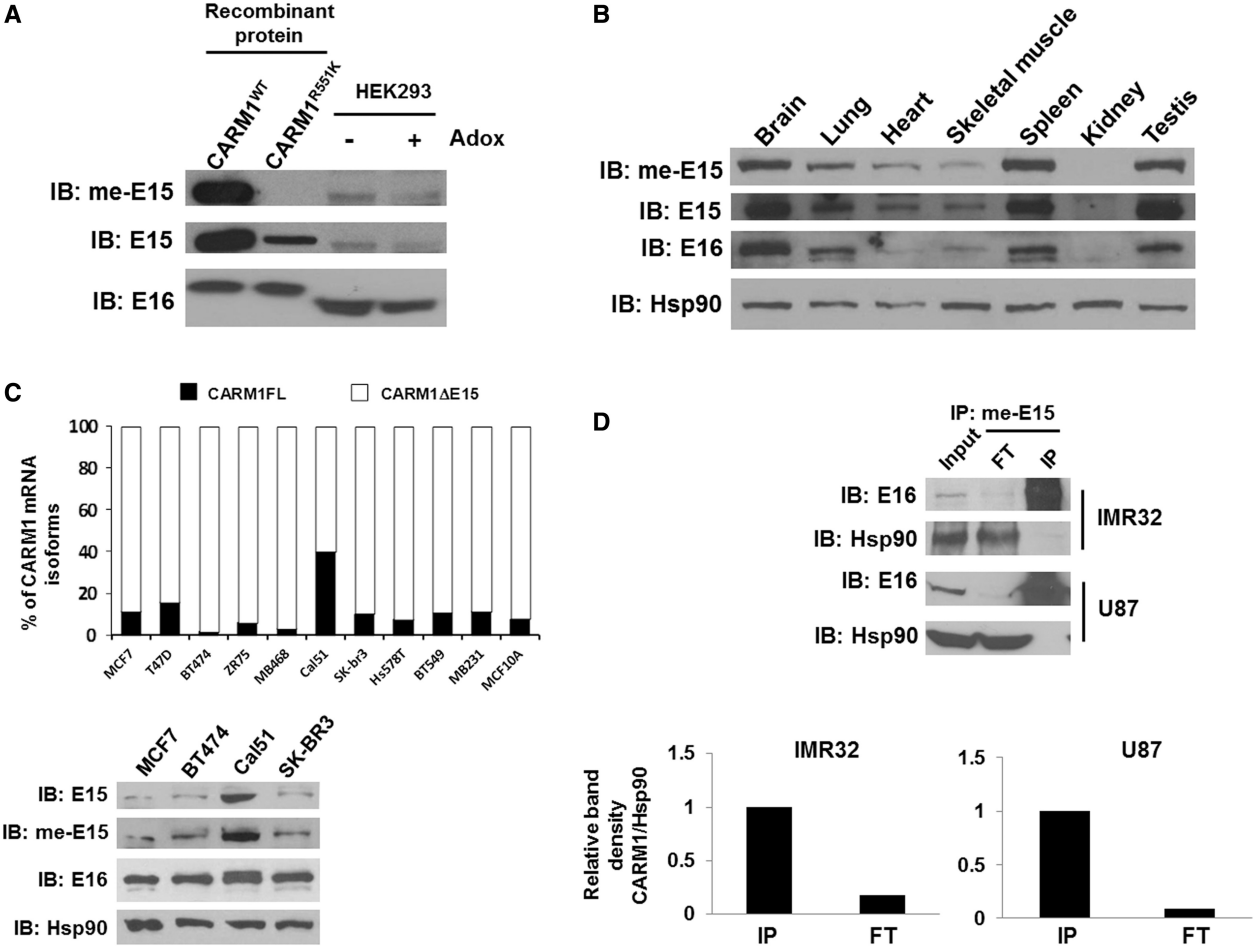
CARM1FL protein, when expressed, is predominately dimethylated. (**A**) me-E15 specifically recognizes dimethylated CARM1 in human cell line. Whole cell lysates were extracted from HEK293T cells treated with or without AdOX, a methylation inhibitor that decreases level of CARM1 automethylation. Total CARM1 and automethylated CARM1 protein levels were determined by western blot using me-E15, E15 or E16, respectively. Recombinant CARM1^WT-FL^ and CARM1^R551K^ proteins were used as positive and negative controls, respectively. (**B**) Automethylated CARM1 and total CARM1 levels in various mouse tissues were detected by western blot using me-E15, E15 or E16, respectively. Hsp90 was used as loading control. (**C**) Top, relative ratios of CARM1 transcripts in various breast cancer cell lines were determined by real-time PCR (*n* = 3). The 18 S was used as an invariant control. Bottom, CARM1FL protein level correlates with the me-CARM1 level in several breast cancer cell lines. Cal51 has the highest level of CARM1FL protein and me-CARM1 in western blot. (**D**) me-E15 can IP nearly all CARM1FL proteins from IMR32 and U87 cells. CARM1 levels detected with E16 in 10% of input, flow-through (FT) and immunoprecipitate (IP) were quantified using Odyssey Imaging System (upper panel). The total CARM1 in input was calculated based on 10% of loaded input and FT and normalized to 1 for input in histograms for IMR32 and U87 cells (lower panel).

We had previously identified a single automethylation site on R551 of recombinant CARM1 (17). The mutation of R551 to K abrogates ERα transcription and pre-mRNA splicing functions, although the enzymatic activity of CARM1 is not affected (17). We took advantage of the me-E15 antibody to examine whether CARM1FL protein expression is sufficient for its automethylation. Figure 3A shows that me-E15 antibody specifically recognizes recombinant CARM1^WT^ but not the automethylation-deficient mutant protein, CARM1^R551K^. Moreover, me-E15 detects the decrease of automethylation of endogenous CARM1 when cells were treated with the methylation inhibitor adenosine dialdehyde (Adox) (20) (Figure 3A). In contrast, E15 antibody also detects CARM1^R551K^, the non-methylated form of CARM1 protein in addition to the activity to detect automethylated CARM1^WT^ (Figure 3A). A western blot probed with me-E15 and E15 shows that meCARM1 mirrors CARM1FL pattern in mouse tissues (Figure 3B). A correlation was also observed among full-length CARM1 transcript level (Figure 3C, top panel), full-length CARM1 protein and methylated CARM1 levels in various cell lines (Figure 3C, bottom panel), implying that cellular CARM1FL protein, when expressed, is predominately automethylated. To further confirm that prevalence of automethylation occurs on nearly all CARM1FL proteins, we immunoprecipitated CARM1 from a glioblastoma cell line U87 and a neuroblastoma cell line IMR-32 using me-E15 and performed a quantitative western blot using the E16 antibody on input and FT fractions. U87 and IMR-32 cells were selected for study because only CARM1FL protein could be detected. Our results showed that me-E15 could deplete nearly all CARM1 by IP measured by western blot and quantification (Figure 3D), suggesting that nearly all CARM1FL proteins are automethylated. Our data strongly suggest that CARM1 automethylation is in large determined by the production of CARM1FL protein, and it ultimately depends on alternative splicing to produce the CARM1FL transcript.

### Differential CARM1 protein isoform distribution in epithelial and stromal cells of normal mouse mammary gland

We next tested the me-E15, E15 and E16 antibodies for IHC to detect endogenous CARM1 expression profiles in normal mouse mammary gland. To optimize the IHC staining protocol using CARM1 antibodies, we generated sections of the formalin-fixed, paraffin-embedded cell pellets using MEF–CARM1+/+ and MEF–CARM1−/− cells. Strong nuclei staining was observed in MEF–CARM1+/+ cells but not in control MEF–CARM1−/− cells with E15 and me-E15 antibodies. The me-E15 immunostaining in MEF–CARM1+/+ cells can be completely blocked with the excess amount of me-E15 blocking peptide, in contrast, the E15 immunostaining was partially blocked with the same amount of me-E15 peptide but was completely blocked with the mixture of two peptides E15 and me-E15 (Supplementary Figure S2). This result suggests that both antibodies are suitable for IHC, and E15 antibody recognizes full-length CARM1 protein but is not methyl specific. Interestingly, the E16 antibody detects CARM1 in nucleus and cytoplasm in MEF–CARM1+/+ cells, and the immunostaining was abrogated by excess amount of E16 peptide.

**Figure 4.**
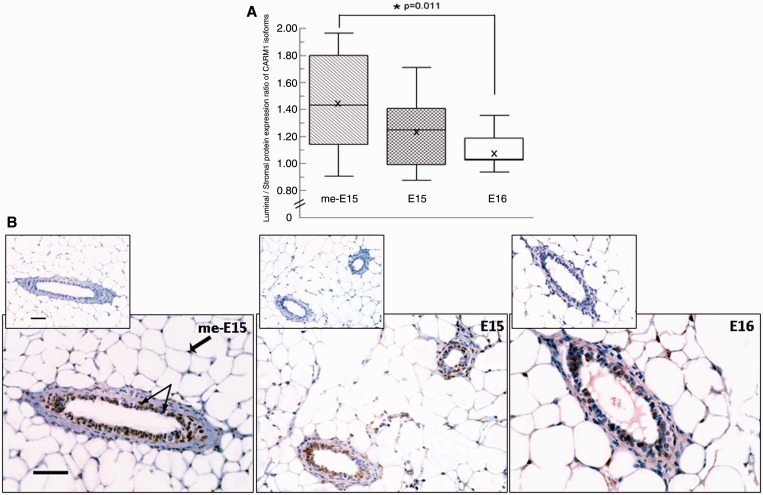
Differential CARM1 isoform distribution in epithelial and stromal cells. Immunohistochemical analyses of luminal:stromal protein expression ratio of CARM1 isoforms in normal mammary glands of young adult mice (*n* = 10–12 mammary glands for each isoform examined). (**A**) CARMFL and CARM1ΔE15 are expressed in both the luminal and stromal compartments of the mouse mammary gland, but automethylation of CARM1 seems to preferentially occur in the luminal (i.e. epithelial) compartment of the mammary gland. ‘x’: mean, *P = 0.01. (**B**) Representative immunohistochemical images of CARM1 isoform distribution in epithelial and stromal compartments of mammary glands of young adult BALB/c mice. Far left panel: thin arrows indicate examples of nuclei staining for CARM1 in cells in the luminal area of the mouse mammary gland, and fat arrow indicates an example of an adipocyte nucleus stained positive for CARM1 within the stroma of the mouse mammary gland. Scale bars: 50 μm. The insets for each panel are immunostaining controls omitting the primary antibody.

Using the refined immunostaining protocol, we examined endogenous CARM1 expression profiles (characterized by luminal to stromal protein expression ratios) in the mouse mammary gland. Consistent with our finding that CARM1FL protein is predominately automethylated, immunostaining with me-E15 and E15 antibodies produced similar expression patterns in luminal and stromal compartments of the normal mouse mammary gland, with the greatest staining in the luminal compartment for both me-E15 (44% greater) and E15 (22% greater; Figure 4). The proportionality of the differences in staining between me-E15 and E15 also suggests that when CARM1 is not automethylated, it tends to be located in the stroma. Further support for the possibility that auto-methylation of CARM1 occurs preferentially in the epithelial compartment of normal mouse mammary glands is the significantly higher luminal to stromal staining ratio observed for me-E15 (automethylated CARM1) compared with E16 (CARM1FL and CARM1ΔE15; *U* = 21, *N*_L_ = 12, *N*_S_ = 10, *P* = 0.011; Figure 4).

### Like CARM1^R551K^, CARM1ΔE15 has impaired co-activator activity to regulate ERα transcription

CARM1 C-terminal domain is required to maintain its full transcriptional activity for ERα (21). Furthermore, we have shown previously that mutation of the auto-methylation site of CARM1 affects its transcriptional activity in reporter assay and ERα target genes (17). To access whether CARM1ΔE15 similarly affects the transcriptional activity as CARM1^R551K^, we performed ERE-luciferase reporter assays in the presence of ERα, GRIP1 and CARM1^WT-FL^, CARM1ΔE15 or CARM1^R551K^. As expected, and similar to CARM1^R551K^ as we previously reported (17), CARM1ΔE15 failed to activate ERα transcriptional activity (Supplementary Figure S3A), whereas the positive control CARM1^WT-FL^ was active. We next examined expression of several endogenous ERα target genes by overexpressing various forms of CARM1. As shown in Supplementary Figure S3B, CARM1^WT-FL^ increases the expression of pS2, IGFBP4 and PTGES in ER18 cells, a HEK293T cell line stably expressing ERα (22). The expression of these genes in CARM1^R551K^ and CARM1ΔE15 transfected cells was identical to that of cells transfected with vector control, suggesting that either mutation of automethylation site or exon 15 deletion to remove the modification site impaired ability of CARM1 to activate ERα-mediated transcription. Western blot results showed that the expression levels of CARM1 proteins are comparable (Supplementary Figure S3C). These results underscore the importance of exon 15 to maintain CARM1 transcriptional co-activator activity and suggest that alternative splicing is the ultimate determinant for regulating C-terminal transcriptional activity of CARM1.

### CARM1FL and CARM1ΔE15 have overlapping yet different substrate methylation activity

**Figure 5.**
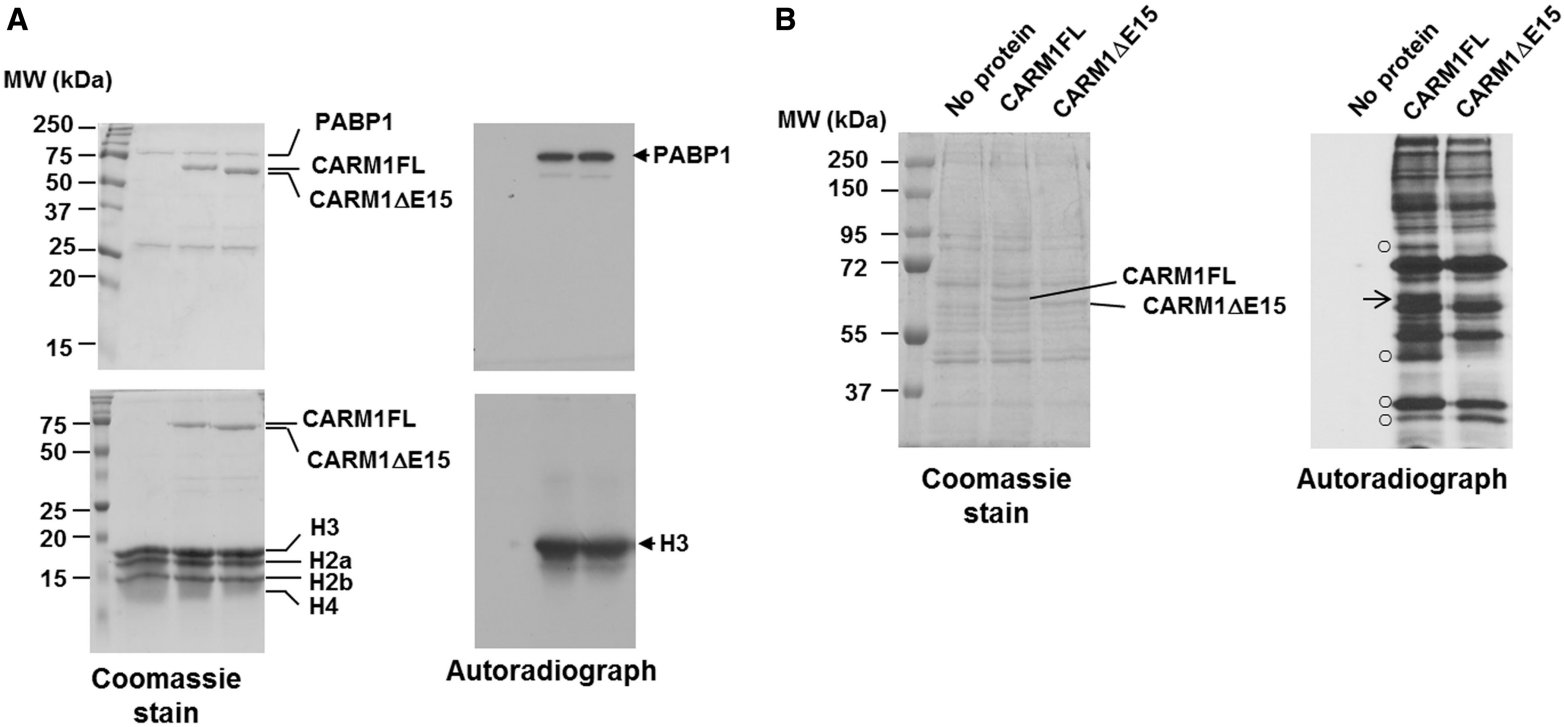
CARM1FL and CARM1ΔE15 have overlapping yet different substrate methylation patterns. (**A**) *In vitro* methylation of PABP1 and histone H3 by recombinant CARM1FL and CARM1ΔE15 proteins (*n* = 2) shows no detectable difference in methyltransferase activities. Coomassie staining (left) and autoradiograph (right) are shown. (**B**) *In vitro* methylation of 10 μg of whole cell lysates derived from CARM1 null MEF cells with ^3^H-SAM and control buffer (no protein), 200 ng of CARM1FL or CARM1ΔE15, respectively. Methylated proteins were shown in autoradiograph. Right arrow: CARM1 automethylation; open circle: differentially methylated proteins.

CARM1 requires its methyltransferase activity for the main cellular functions *in vivo* (23). Previously, we found that mutation of the automethylation site of CARM1 has no effect on its methyltransferase activity (17). To determine whether exon 15 deletion of CARM1 affects its ability to methylate cellular substrates, we performed *in vitro* methylation assays using recombinant CARM1 proteins and the known CARM1 substrates PABP1 or histone H3. Figure 5A shows that the activity of CARM1FL and CARM1ΔE15 to methylate these substrates is indistinguishable. Next, we examined the global substrate methylation activity of CARM1FL and CARM1ΔE15 proteins toward the CARM1 null mouse embryonic fibroblast cell lysates. Surprisingly, even when total lysate proteins were separated on a 1D SDS–PAGE, the autoradiograph shows that although the overall substrate methylation pattern is similar, CARM1FL notably methylates more substrates than CARM1ΔE15 (Figure 5B, open circle). As expected, CARM1 automethylation is only detected with CARM1FL and not in CARM1ΔE15 (Figure 5B, right arrow). This result implies that in addition to sustaining R551 automethylation, exon 15 encompasses a necessary sequence for mediating interaction of CARM1 with some protein substrates.

## DISCUSSION

### Protein arginine methylation level may be controlled by alternative splicing

We have previously shown that automethylation of CARM1 plays an essential function in coupling of transcription and splicing events (17). Although the mass spectrometry data demonstrated that nearly 100% of the recombinant CARM1 is automethylated, it remains unknown whether endogenous CARM1 is automethylated and how automethylation is regulated. To address these questions, we developed a peptide antibody that specifically recognizes the automethylated CARM1 (me-E15Ab) and showed that endogenous CARM1 in cell lines and tissues is automethylated to nearly completion (Figure 3). Because protein arginine demethylase has not been firmly identified, the high efficiency of automethylation of CARM1FL protein implies that, rather than via demethylation, alternative mechanism(s) may be used to regulate the level of protein arginine methylation. Our findings raise an interesting possibility that CARM1 methylation of protein substrates, as well as itself, may be controlled at the level of alternative splicing. One example supporting this notion is PABP1, whose splice variant lacks amino acids 447–535 encompassing the methylation site by CARM1 (R455, R460) (24). Interestingly, other PRMT substrates also frequently have missing exons encompassing the site(s) of methylation by PRMTs. For example, two PRMT1 substrates, the SAM68 isoform 2 missed a region (amino acids 37–71) containing the methylation sites R45 and R52 (25) and the hnRNP A1 isoform 2 missed a region (amino acids 203–307) containing multiple methylation sites (26) by PRMT1. Further, MBP, a PRMT5 substrate, exists in multiple isoforms, among which isoform 2 (missing amino acids 193–304) and isoform 6 (missing amino acids 240–250) lack the methylation site (R241) by PRMT5 (27). All of the current evidence supports that alternative splicing of transcripts may be the underlying, yet under-appreciated, mechanism for regulation of cellular protein arginine methylation levels by PRMTs.

### CARM1ΔE15 may have distinct biological functions from CARM1FL in breast cancer

CARM1ΔE15 seems to be the predominant isoform in cancer cell lines (Figure 1B). Moreover, our data suggest that the ratio of CARM1ΔE15/CARM1FL may be higher in cancer cell lines as compared with immortalized cell lines with moderate tumorigenic potential (28). This correlation implies that CARM1ΔE15, the main isoform in cancer epithelial cells, may have distinct functions from those of CARM1FL. The two isoforms of CARM1 transcripts and the resulting proteins may explain the functional discrepancy in the literature: some studies have shown that CARM1 is involved in cancer cell proliferation (9,10), whereas we showed that overexpressing full-length mouse CARM1 cDNA resulted in increased differentiation and decreased proliferation (8). The contradictory reports of CARM1 function could attribute to different isoforms in separate studies. We showed that the two isoforms displayed differential methylation patterns (Figure 5B). Further studies are needed to elucidate whether CARM1FL and CARM1ΔE15 elicit functional discrepancy.

### CARM1 alternative splicing is conserved across species

Using antibodies recognizing full-length (E15) and the two CARM1 isoform proteins (E16), we showed that alternative splicing of CARM1 transcripts is required for generating two isoform proteins, and alternative splicing is evolutionally conserved in mouse, rat and human and across various tissues. Although this study focused on the two human transcripts that are annotated in emsembl.org and encode CARM1FL and CARM1ΔE15 proteins, four CARM1 transcripts with variations in the C-terminus were previously described by Ohkura *et al.* (19). The study by Ohkura *et al.* (19) analyzed four CARM1 transcripts of rat origin derived from the primary cultured rat forebrain cells; however, it is unknown whether all these transcripts encode functional proteins. The CARM1-v1 and v4 transcripts described by Ohkura *et al.* (19) correspond to CARM1FL and CARM1ΔE15 in our study. Ohkura *et al.* showed that the extent of CARM1-v1 activity was similar to that of CARM1-v4 activity in an ERE-luciferase reporter assay. This conclusion is contradictory to our finding that CARM1ΔE15 had reduced activity in the reporter assay as compared with CARM1FL (Supplementary Figure S3). However, we did not clone CARM1 transcripts of rat origin to directly compare with the two human transcripts in luciferase reporter assay, the discrepancy in the reporter activity could be due to different cell lines used for transfection assay (COS-7 cells were used by Ohkura *et al.*, and HEK293T cells were used in this study), different ERE-luciferase reporter constructs or the species-specific difference at the transcript level for CARM1. We cannot exclude the possibility that additional CARM1 isoforms exist in different rat strains possibly used in the Ohkura *et al.*’s (19) article because the origin of the rat forebrain cells was not specified. Nonetheless, we are confident that at least in the ACI rat strain used in our study (Figure 1D), only two major CARM1 isoforms can be detected using primers amplifying the region encompassing exon 11–16.

### CARM1 isoforms in normal mouse mammary gland and possible link to ERα biology

In normal breast tissue, CARM1 regulates ERα-stimulated proliferation and differentiation, activities which are deregulated in breast cancer, with the balance shifting to enhanced ERα proliferative activity (8,29). The present study demonstrates that the two endogenous CARM1 isoforms, CARM1FL and CARM1ΔE15, expression patterns differ in normal mouse mammary gland. Although both isoforms are expressed in luminal and stromal compartments, CARM1FL is preferentially expressed in the luminal (epithelial) area of normal mouse mammary glands. The preferential expression of the ERα activity mediating isoform CARM1FL in the epithelial luminal cells may be expected, as we have shown previously that ERα protein levels in normal mouse mammary gland is significantly higher in epithelial (luminal) than in stromal areas (30), and CARM1 levels have been correlated to ERα levels, albeit in ERα-positive tumors (8). Moreover, and in support of our *in vitro* data, our results show that the automethylated CARM1FL is the predominant isoform in the luminal compartment of normal mouse mammary glands. Taken together, these data suggest that alternative splicing events differ between epithelial and stromal cells to cause the preferential expression of CARM1FL and CARM1ΔE15, respectively, and that there may be epithelial-based mediators of the automethylation of CARM1FL. Furthermore, as we have shown in the present study that CARM1ΔE15 is unable activate ERα transcriptional activity, and CARM1ΔE15 is preferentially expressed in the stroma, the data suggests different roles for ERα in the epithelium and stroma. Further studies are needed to elucidate the functional significance of the distribution of the CARM1 isoforms and the factors that may be mediating the exon retention within the epithelial compartment of the mammary gland.

## SUPPLEMENTARY DATA

Supplementary Data are available at NAR Online: Supplementary Figures 1–3.

## FUNDING

National Institutes of Health [RO1CA125387]; National Scientific and Engineering Research Council (NSERC) of Canada [Grant #288318 to L.K.]; Trent University Internal Research Grants (to L.K.); DOD ERA of HOPE Scholar Award [Grant #W81XWYH-11-1-0237 to W.X.]; Villas Associate Award (to W.X.); AOF (to C.G.C.); Royal Thai Government Scholarship (to P.C.). Funding for open access charge: DOD grant [W81XWYH-11-1-0237 to W.X].

*Conflict of interest statement.* None declared.

## Supplementary Material

Supplementary Data
