# Supplementary material for: CARM1 automethylation is controlled at the level of alternative splicing
Source: Nucleic Acids Res. 2013 May 30;41(14):6870–80. doi: 10.1093/nar/gkt415 (PMC3737532; doi:10.1093/nar/gkt415)
Supplement: Supplementary Data [file supp_41_14_6870__index.html]

CARM1 automethylation is controlled at the level of alternative splicing — CARM1 automethylation is controlled at the level of alternative splicing — Supplementary Data 

# CARM1 automethylation is controlled at the level of alternative splicing

## Supplementary Data

files

**Files in this Data Supplement:**

- Supplementary Data - pdf file
