# Supplementary material for: CARM1 automethylation is controlled at the level of alternative splicing
Source: Nucleic Acids Res. 2013 May 30;41(14):6870–80. doi: 10.1093/nar/gkt415 (PMC3737532; doi:10.1093/nar/gkt415)

Figure legends for supplementary figure:

Supplementary Figure 1: CARM1FL and CARM1ΔE15 could form dimers in solution. MEF-CARM1 -/- cells were co-transfected with either GFP-CARM1FL and Flag-CARM1FL or GFP-CARM1FL and Flag-CARM1ΔE15, respectively. Cell lysates were harvested 48 hours after transfection. GFP-CARM1FL was immunoprecipitated using GFP antibody, and the interacted Flag-CARM1 isoform proteins were detected using E16 antibody.

Supplementary Figure 2. Validation of CARM1 antibodies for immunohistochemical staining. Both control MEF (MEF-CARM1+/+) and CARM1 knockout MEF (MEF-CARM1-/-) cells were seeded on the poly-L-Lysine slides to 70% confluence. The cells were then fixed and IHC staining was performed using primary antibodies E16 (1:250), me-E15 (1:250), or E15 (1:250) at indicated dilution. To determine the specificity of Ab for immunostaining, 5 μg/ml of blocking peptides were used to pre-incubate with each antibody before IHC. The experiment was performed for at least three times and the representative photograph was shown.

Supplementary Figure 3. CARM1ΔE15 and $CARM1^{R551K}$ have impaired co-activator activity in ERα-dependent transcription. (A) ERE-luc reporter assay in CARM1FL, CARM1ΔE15 and $CARM1^{R551K}$ transfected HEK293 cells. (B) E2-dependent ER target gene induction is impaired in CARM1ΔE15 or $CARM1^{R551K}$ transfected ER18 cells, a HEK293T cells stably expressing ERα. Cells were seeded in 24-well plate, and

transfected with 200 ng of pCMX-Flag vector, pCMX-Flag-CARM1FL, pCMX-Flag-CARM1ΔE15, or pCMX-Flag-CARM1^{R551K}, respectively. 24 hours later, the cells were treated with 10 nM E2 and the mRNA levels of pS2, IGFBP4 and PTGES were determined by real-time PCR and normalized by 18S invariant control. CARM1 protein level of each sample was detected by western blot, β-Actin was used as invariant control. Data are mean ± SD of three independent experiments. ** P<0.01, vs cells transfected with the control vector. (C) Transfected cell lysates were resolved by SDS-PAGE and detected with E16 and anti-Flag antibodies. Western blot results showed similar expression levels of CARM1^{WT-FL}, CARM1ΔE15 and CARM1^{R551K}.

Supplementary Figure 1

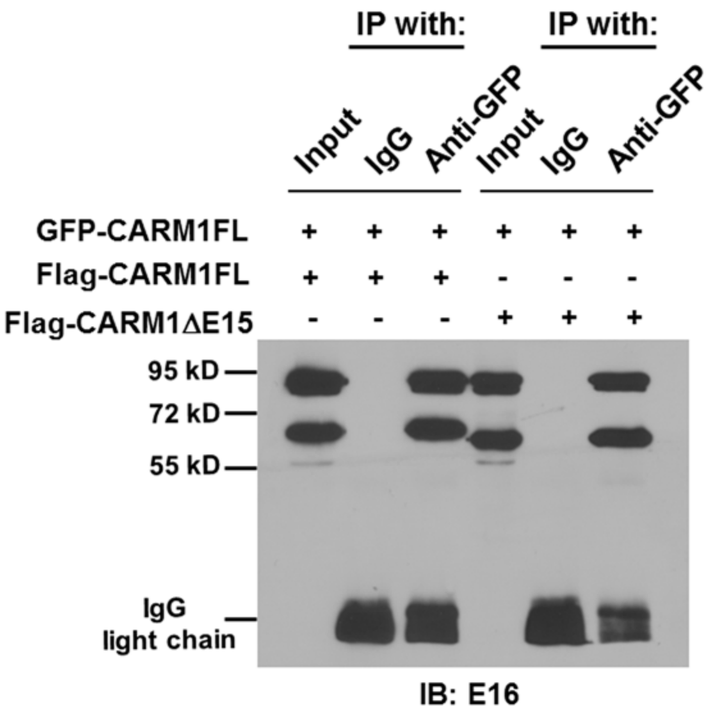

Supplementary Figure 2

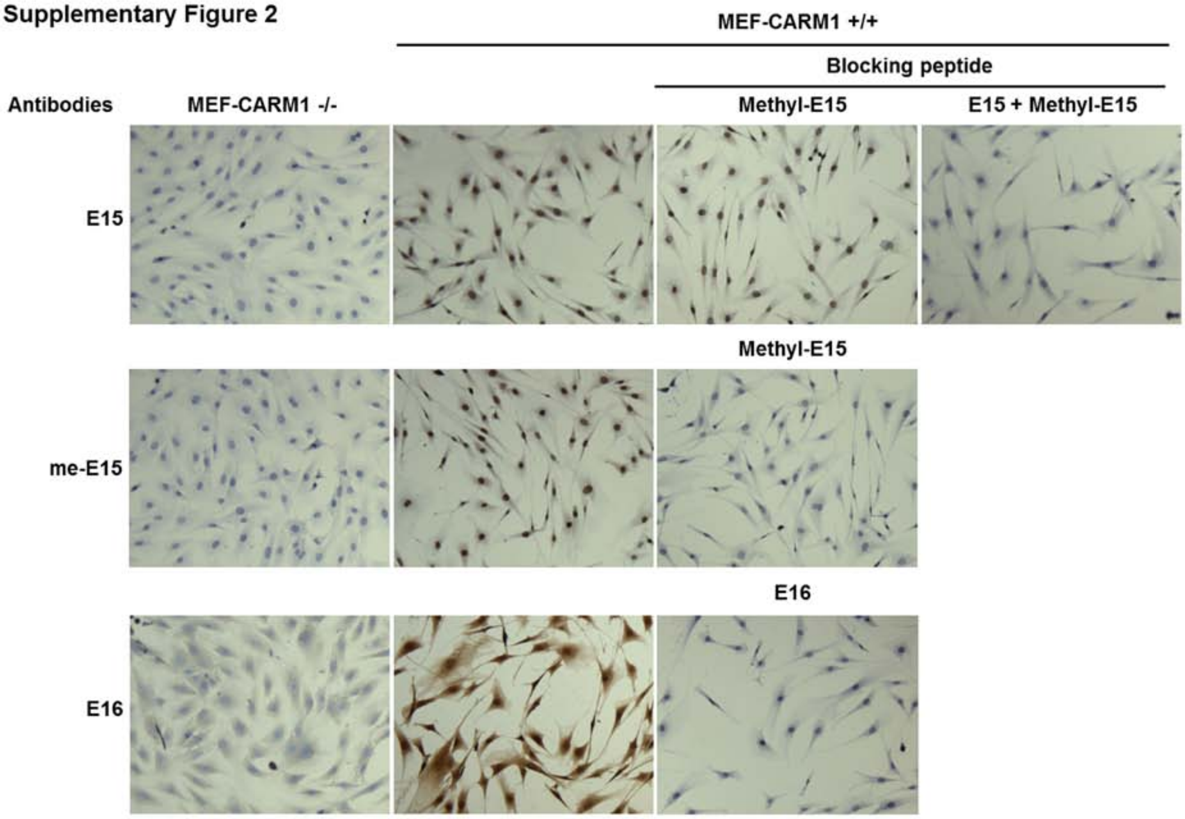

Supplementary Figure 3

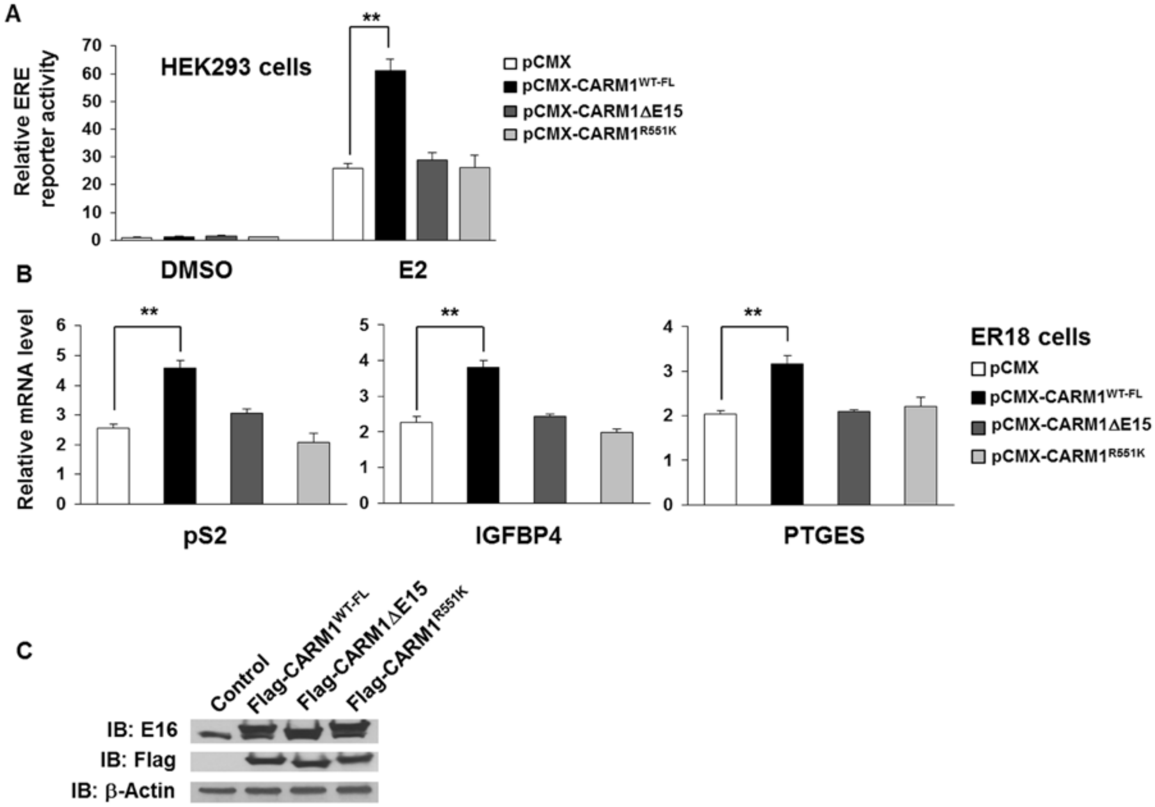

Supplement: Supplementary Data [file supp_gkt415_nar-00204-m-2013-File007.pdf]
